# Towards human exploration of space: The THESEUS review series on immunology research priorities

**DOI:** 10.1038/npjmgrav.2016.40

**Published:** 2016-12-01

**Authors:** Jean-Pol Frippiat, Brian E Crucian, Dominique J-F de Quervain, Daniela Grimm, Nicola Montano, Siegfried Praun, Benno Roozendaal, Gustav Schelling, Manfred Thiel, Oliver Ullrich, Alexander Choukèr

**Affiliations:** 1 Stress Immunity Pathogens Laboratory, EA7300, Lorraine University, Nancy, France; 2 NASA Johnson Space Center, Houston, TX, USA; 3 Departments of Medicine and Psychology, University of Basel, Basel, Switzerland; 4 Department of Biomedicine, Pharmacology, Aarhus University, Aarhus, Denmark; 5 Cardiovascular Neuroscience Laboratory, Department of Biomedical and Clinical Sciences, University of Milan, Milan, Italy; 6 VF Services GmbH, Absam, Austria; 7 Department of Cognitive Neuroscience, Radboud University Medical Centre and Donders Institute for Brain, Cognition and Behaviour, Radboud University, Nijmegen, The Netherlands; 8 Department of Anaesthesiology, ‘Stress and Immunity’ Laboratory, University of Munich, Munich, Germany; 9 Department of Anaesthesiology and Surgical Intensive Care Medicine, University Medical Center Mannheim, Medical Faculty Mannheim, University of Heidelberg, Heidelberg, Germany; 10 Institute of Anatomy, Faculty of Medicine, University of Zurich, Zurich, Switzerland

## Abstract

Dysregulation of the immune system occurs during spaceflight and may represent a crew health risk during exploration missions because astronauts are challenged by many stressors. Therefore, it is crucial to understand the biology of immune modulation under spaceflight conditions in order to be able to maintain immune homeostasis under such challenges. In the framework of the THESEUS project whose aim was to develop an integrated life sciences research roadmap regarding human space exploration, experts working in the field of space immunology, and related disciplines, established a questionnaire sent to scientists around the world. From the review of collected answers, they deduced a list of key issues and provided several recommendations such as a maximal exploitation of currently available resources on Earth and in space, and to increase increments duration for some ISS crew members to 12 months or longer. These recommendations should contribute to improve our knowledge about spaceflight effects on the immune system and the development of countermeasures that, beyond astronauts, could have a societal impact.

## Introduction

Spaceflight is a unique stress model impacted consistently or intermittently by a myriad of stressors of psychosocial and physical origin, high G forces at the time of launch and landing, increased radiation, sleep deprivation and persistent circadian misalignment, microgravity, and nutritional factors while in space. This multitude of factors has profound immune modulatory effects on humans and animals and can lead to certain types of immune dysregulation with compromised defenses against infections, thereby representing a potential barrier to long-term space exploration (for more information consult recent reviews^1–4^). For example, 15 of the 29 Apollo astronauts contracted bacterial or viral infections either during the mission or within a week of returning.^5^ Moreover, recent results after mission to the International Space Station revealed that during spaceflight some aspects of human immunity may be oversensitized, as evidenced by some astronauts who experienced prolonged allergic/skin hypersensitivity symptoms.^6,7^

Several studies have shown that spaceflight conditions induce frequently hypoplasia of the spleen in rats and mice.^8,9^ Variations in peripheral blood leukocyte subsets were also reported at landing. These modifications could be mediated by changes in the expression of adhesion molecules^10,11^ and/or the redistribution of body fluids in microgravity.^12^ An increase in the number of peripheral blood neutrophils has been often observed. Results for other cell types are variable thereby revealing the sensitivity of immune cell populations to differences in spaceflight conditions, post-flight procedures, environmental influences, and experimental designs.

In the same way, it has been shown that the phagocytic and oxidative functions of neutrophils are affected by spaceflight conditions.^13,14^ Astronauts’ monocytes exhibit phenotypic and cytokine-production deregulations and a reduced ability to engulf *E. coli*, elicit an oxidative burst and degranulate.^14–16^ The response of astronauts’ monocytes to Gram-negative endotoxins is modulated by spaceflight-associated factors.^17^ Low natural killer cell cytotoxicity and a delay in responses to hypersensitivity skin tests were also observed.^18–20^ Moreover, reactivation of latent herpes viruses (e.g., varicella zoster virus, cytomegalovirus, and Epstein-Barr virus) has frequently been reported and is correlated with a drop in interferon production and elevated levels of stress hormones, suggesting that spaceflight-associated stressors may be responsible for these reactivations.^21–28^ Latent virus reactivation can therefore be considered as a good biomarker of spaceflight-induced weakening of cell-mediated immunity.

Similarly, the activation of T lymphocytes is severely depressed under low-gravity conditions as highlighted by Cogoli’s team, which performed many investigations on this topic following their discovery of this phenomenon in 1984.^29–33^ This lower response has several explanations. First, changes in gene expression as lower expressions of Interleukin-2 (IL-2) and IL-2 receptor were observed under real and simulated microgravity^31–40^ and it was shown that gravity can regulate T-cell activation by blocking translation via noncoding microRNA.^41^ Second, reduced cell–cell interactions and cytoskeleton structure changes. Indeed, T lymphocytes motility was found to be affected, the motility of monocytes was severely reduced and the structure of their cytoskeleton was modified.^30,34,40,42–46^ These motility and cytoskeleton changes could reduce the interaction between T lymphocytes and monocytes, essential for delivering the costimulatory signal. Because the cytoskeleton is involved in signal transduction^47^ and could be the structure through which cells sense gravity,^48^ inhibition of T-cell response could thirdly result from alterations in signaling events. Hughes–Fulford’s group analyzed differential gene expression in Concanavalin A and anti-CD28-activated human T cells^36^ and discovered that the impaired induction of early genes regulated primarily by transcription factors NF-κB, CREB, ELK, AP-1, and STAT1 contributes to T-cell dysfunction under altered gravity. They also showed that the protein kinase A (PKA) signaling pathway is downregulated under microgravity. As NF-κB, AP-1, and CREB are all regulated by PKA, these findings indicate that PKA is a key player in gravity-mediated modulation of T-cell activation. In accordance with these results, a recent study confirmed that the Rel/NF-κB signaling pathway and the transcription of key immediate early genes involved in T-cell activation are inhibited by microgravity.^49^ Fourth, the disturbed expression of cell cycle regulatory proteins^50^ and the induction of apoptosis^51,52^ could also contribute to impaired T-cell activation in microgravity, whereas key proteins of T-cell signal modules seemed not to be severely disturbed in microgravity.^53^ Finally, post-flight cytokine data collected from crew members^54^ revealed a decrease in TH1 cytokine expression, which can contribute to decreased natural immunity and suggests a TH2 cytokine shift. This TH2 shift represents a significant clinical risk for TH2-related autoimmune diseases, disease susceptibility related to diminished cell-mediated immunity, as well as allergies and hypersensitivities as recently demonstrated.^6,7^ Although a well-characterized post-flight phenomenon, immune dysregulation occurs, and persists during spaceflight, confirming in-flight dysregulation distinct from the influences of landing and readaptation following deconditioning.^55,56^

Humoral immunity has rarely been investigated in astronauts. According to the few studies available, no significant changes in plasma levels of immunoglobulin were observed after short-term spaceflights,^10,14,57^ but inconsistent results were reported after long-duration missions. Konstantinova *et al.*^58^ reported increased levels of serum immunoglobulin, particularly total IgA and IgG, whereas Rykova *et al.*^14^ indicated that the total amounts of serum IgA, IgG, and IgM were unchanged after prolonged missions. Furthermore, nothing is known about B-cell activation under spaceflight conditions. Only studies performed using ground-based analogs have been reported.^32,59^ Studies performed with the urodele amphibian *Pleurodeles waltl* as animal model confirmed the increase of neutrophils in peripheral blood at landing.^60^ As for astronauts, this increase can be attributed to stress because it was shown that neutrophilia occurs in the urodele amphibian *Notophthalamus viridescen*s when it is treated with hydrocortisone or adrenocorticotropic hormone (ACTH), two stress hormones, or when it is subjected to environmental stress.^61^ Amphibians are interesting models for analyzing the effects of spaceflight on the immune system because cardinal elements of the adaptive immune system are shared by all gnathostomes.^1,62–69^ Later on, adult *P. waltl* were immunized with protein onboard the Mir space station. The analysis of these animals revealed an increase of IgY heavy chain mRNAs (IgY is the physiological counterpart of human IgA^66^) in the spleen of flown animals.^70^ This increase supports previous observations made in astronauts by Konstantinova *et al.*^58^ Furthermore, these animals allowed demonstrating that spaceflight affects antibody production in response to an antigenic stimulation. Indeed, the use of the different VH gene subgroups^69^ and the expression of individual VH gene segments^71^ were observed to be modified under spaceflight conditions. In addition, these animals enabled, for the first time, the demonstration that somatic hypermutations, which diversify antibody binding sites to improve their affinity, occur in space following immunization but at a lower frequency.^72^ This observation suggests that antibody affinity maturation could be less efficient in space, thereby decreasing the efficiency of the immune response. Finally, it was noted that the transcription levels of IgM heavy chains and of an early B-cell transcription factor are modified when *P. waltl* embryos are subjected to gravity changes, suggesting a modification in lymphopoiesis.^73^ This hypothesis was then confirmed in mice subjected to ground-based models.^74,75^

**Figure 1:**
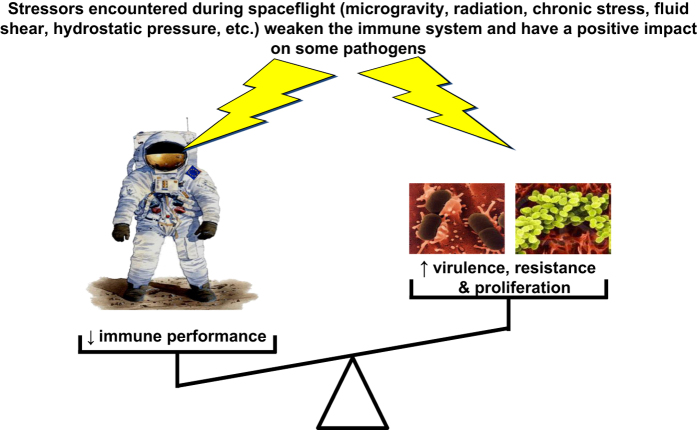
Numerous dysregulations of the immune system have been reported during and following spaceflight. In parallel, spaceflight has been shown to increase the virulence, antibiotic resistance, and proliferation of some pathogens suggesting that susceptibility to infections could be enhanced during space missions.

In parallel, changes in microbial growth characteristics and pathogenicity have been observed for several microorganisms during spaceflight.^76–80^ These data, coupled to dysregulations of the immune system, suggest that opportunities for microbes to establish foci of infection could be enhanced during space missions (Figure 1). Furthermore, some data suggest that antibiotics could be less effective in space.^81^

**Figure 2:**
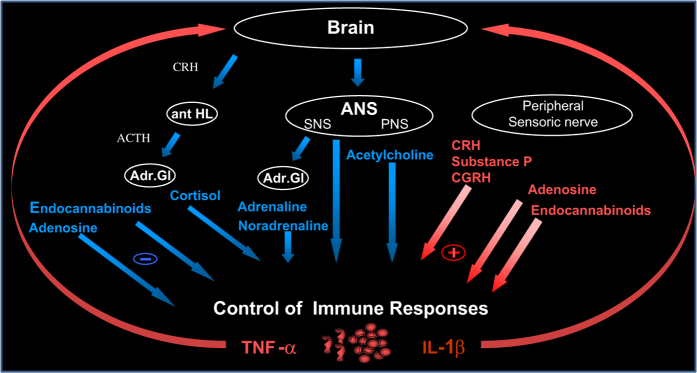
Interlinks between the central nervous system and the immune system. ANS, autonomic nervous system; IL, interleukin; PNS, peripheral nervous system; SNS, sympathetic nervous system; TNF-α, tumor necrosis factor α.

Finally, it was shown that spaceflight induces significant changes in the thymic mRNA expression of genes that regulate stress and glucocorticoid receptor metabolism.^82^ To better approach the consequences of physical and emotional stressors in man exposed to spaceflight, investigations have been undertaken by applying complementary and elaborated blood, urine and saliva sampling, and analyses, together with questionnaires based emotional stress monitoring. These investigations in man ranged from very acute gravitational challenges (parabolic flight) to short (1–2 weeks) and long (4–7 months) duration ISS missions and showed that there is a shift from an alert state of primed innate immune cells after acute gravitational stress to a decrease of their activity after spaceflight. Adrenocortical stress-responses suggest that sympathetic nervous system (SNS) responses predominate after short spaceflights, although long flights are characterized by glucocorticoid-mediated changes. Furthermore, central activation of SNS, even via non-adrenomedullary pathways, such as sleep deprivation,^83^ has recently been shown to have a pivotal role in the regulation of inflammation^84^ and innate immunity.^85^ The immunotropic endocannabinoid system is also activated under acute gravitational stress and in space.^86^ Further, Earth-bound studies have provided evidence of emotional stress-sensitive immune changes in human.^54,86–88^ These data highlight the complex systemic compromise of the immune system in response to exposure to multiple stressors during spaceflight (Figure 2).

Altogether, these data indicate that spaceflight-induced alterations of the immune system have the potential to be a serious clinical risk to astronauts and cosmonauts during exploration-class deep space missions beyond Earth orbit.^3,89^ This represents an area that should be considered more thoroughly, via both ground-analog studies and investigations onboard the ISS, to ensure long-term survival in space stations and sustain habitation during future missions to the moon, Asteroids, or on any planet i.e., Mars.

## Identified key issues

Consequently, experts working in the field of space immunology and experts working in related areas (e.g., psychoneuroendocrine and autonomous nervous system regulation) established a questionnaire that was sent to scientists around the world to identify key issues that should be addressed for the preparation of future long-duration space missions. The analysis of collected answers highlighted the following points:

- The identification and the quantification of stress factors encountered during a space mission and their impact on the immune system should be better studied.
- The extension of ISS increments to 1 year or longer is of importance to address the following questions: (i) are immune system development, response, and regulation as efficient in space (ISS/moon/Mars) as on Earth? (ii) What are the consequences of chronic immune changes on disease during and after long-duration missions? (iii) What are the consequences of long-duration (⩾1 year) missions on the degree of immunosuppression/modulation? (iv) What effects Lunar or Mars dusts, habitat environment and other chemicals, have on immune system performance?
- Are stress-dependent virus reactivation patterns linked to cancer development?
- The interaction between the immune system and other stress-sensitive systems (neurophysiological and others) should be further studied.
- The definition and testing of countermeasures.

In order to understand and track the footprint of space exploration on the immune system, and its interactions with other organs, it appears crucial that these key issues are addressed through an appropriate technical and structural framework. In this context, three interlinked approaches emerged: (i) pathophysiological cellular pathways and complex molecular mechanisms analysis using isolated cell systems. (ii) Implementation of animal experiments to investigate the compromise of the immune system after exposure to multiple stressors encountered during spaceflight. (iii) Coordinated investigations in man to better approach the consequences of multifactorial effects of physical stressors and emotional stress induced by spaceflight.

## Knowledge gaps and research needs identified by the online survey

In light of future manned spaceflight for exploration, extension of ISS increments for some ISS astronauts/cosmonauts to one year or longer has been considered by all Expert Group (EG) members, and the majority of investigators participating to the online consultation, of very high importance. It is fortunate that the first 1-year ISS expedition has been successfully accomplished and may pave the way for further repeat or an even further prolongation.

The development of inflight hardware to perform easy-to-handle and noninvasive exams to monitor immune and hemostatic changes was considered to be of very high importance by EG as well as by 90% of the consulted specialists. Indeed, up to now, due to technical constrains and limited astronaut time dedicated to each experiment, most inflight assessments resulted from the analysis of inflight fixed cells.

To achieve a higher relevance of gathered data, especially for critical investigations, a need for (i) repeating inflight experiments at least twice and (ii) some standardization of experiment design, methods, assays, and so on is requested. Both points were considered of high or very high importance by >90% of consulted scientists.

Before going to space, test and control experiments should be performed using the full scale of Earth-bound models that can adequately simulate specific spaceflight conditions. Fifty percent of the respondents considered this point as very highly relevant and 40% as highly relevant. The appropriate terrestrial analog may vary based on the space-physiological system of interest. For immune dysregulation Arctic, undersea, and Antarctica deployment have all be demonstrated to have similarities to flight. Prolonged head-down tilt bed rest can also be used. However, conflicting results have been reported using this model; some studies reported limited changes in the immune system,^90,91^ whereas others reported changes in cytokine production, immune cell activation and innate immunity.^92–97^

The establishment of a bank of tissue and plasma samples available to the scientific community was defined by the majority of EG members and external experts (>90%) to be very important to extract maximal information as well as to reanalyze samples when new tools become available.

Taking advantage of health data files from ISS crew members, with retrospective and prospective access, is necessary to estimate clinically relevant changes in regard to immune alterations.

## Proposed investigations and recommendations

Given these knowledge gaps and research needs, the expert group established a list of recommendations:

- The immune system is a target and a sensor of environmental changes affecting the organism as a whole. Therefore, interdisciplinary approaches are required to understand how homeostasis and immune functions are affected through other organs under acute or chronic stress conditions, both in space and on Earth.
- Multidisciplinary approaches should include the full-scale use of available *in vitro* and *in vivo* methods using rodents or other animal models, as well as clinical and space related Earth-bound studies.
- It is highly recommended to standardize some methodologies used for immune research as well as repeating space experiments.
- The establishment of an all ISS-partners international biological sample archive (samples collected from space crew members and animals) is mandatory to allow the extraction of a maximum of information from these samples. Such a sample archive would be most beneficial if multiple types of biological samples, such as saliva, urine, plasma, and blood cells could be appropriately preserved for various soluble, cellular and/or molecular analyses.
- The access to anonym health data from crew members, retrospectively and prospectively, should be allowed to complete space experiments results.
- The development of new technologies to diagnostic immune changes in minute amounts of blood should be encouraged. Preliminary efforts to develop such microgravity-compatible laboratory instruments have been initiated both in Europe and in North America and should be continued because such hardware would be a benefit for space and Earth medicine.

## Trans- and cross-disciplinary aspects

The understanding of stress related immune challenges in space is of high relevance to understand the biology of cancer, immunology and inflammation in astronauts as well as in the young and ageing population on Earth. The translational aspects of technological developments for space research constitute also a potentially highly attractive spin-off for, e.g., the non-invasive monitoring of immune and health conditions in minute amounts of blood in space and on Earth. Finally, the immune system is one of the largest and most widespread systems with hormonal and neuronal connections (Figure 2). The immune system is strongly linked with almost every organ homeostasis and can be affected, together with other systems, upon stress challenges. As an example, stress induces the secretion of hormones which can modulate immune responses and also promote bone turnover. Moreover, immune responses are susceptible to exercise and nutritional factors as well as to environmental factors like radiation, the microbial load and the oxygen tension in the habitat. Therefore, there are manifold non-selective interactions with other fields and topics such as bone, muscle, kidney, lung, neurophysiology, cognitive performance, nutrition, exercise, the cardiovascular system, habitat environment and design, radiation, and health (prevention, diagnosis, and therapy). Consequently, several types of countermeasures could be tested that affect directly or indirectly the immune system such as vaccination, nutrition, exercise but also pharmacological and psychosocial approaches dedicated to reducing stress because it is an important contributor to immune dysfunction. These countermeasures are also interesting for other conditions in which the function of the immune system is compromised on Earth such as people subjected to acute or chronic stress.

## Conclusion and Earth benefits

It is an international scope to gather more knowledge on how the immune system is webbed in the pathology and clinical appearance of disease, from it cellular functions to an orchestrated action of immune functions in the complex human system. In the fields of fundamental and clinical immunology, the analyses of the links with other organs are important to address societal questions such as aging and the consequences of a sedentary life style. From clinical investigations in the healthy and the sick, the lifesaving importance of adequate immunity at the switch between the host and the environment was never clearer before. On-orbit immune dysregulation has the potential to be of either a “hyper” or a “hypo” nature, or even both as various innate and adaptive cellular components may respond differently during flight. Beside controlling infections and eliminating germs, immunological responses are also capable of eliminating non-functional or dysfunctional cells, e.g., tumor cells. Failure to establish adequate immune answers and to distinguish between ‘self’ and ‘non-self’ can result in autoimmune diseases (e.g., rheumatoid arthritis), acute and life-threatening infections and overwhelming systemic immune responses (e.g., septicemia) or the development of cancer. Changes to this endogenous balance between the immune and other systems due to psychological or environmental factors can impact this finely balanced, endo-, para-, and autocrine-controlled immune function, resulting in immune dysfunction and disease. The understanding of these deregulations, together with the development of appropriate countermeasures, can help space travelers and people on Earth.
